# The Impact of Next Generation Sequencing in Cancer Research

**DOI:** 10.3390/cancers12102928

**Published:** 2020-10-12

**Authors:** Katia Nones, Ann-Marie Patch

**Affiliations:** QIMR Berghofer Medical Research Institute, Brisbane, 4006 Queensland, Australia; Ann-Marie.Patch@qimrberghofer.edu.au

Next generation sequencing (NGS) describes the technical revolution that enabled massively parallel sequencing of fragmented nucleic acids, thus making possible our current genomic understanding of cancers. It took an international consortium, using factory-like centers and high-throughput Sanger (terminator dye sequencing), more than 10 years to release the completed reference human genome in 2003. The generation of this high quality human genome reference and associated gene model annotation was essential for cancer genomics studies. However, the scale of resources required to generate the human genome reference highlighted the need for innovative sequencing approaches that would redefine the term “high-throughput sequencing”. NGS is the term used for the new form of sequencing that emerged in the mid-2000s and is the product of competing technologies that led to increased speed, capacity, accuracy of sequencing machines and the progressive fall in the cost of sequencing per base pair. It is this sequencing revolution which enabled researchers to conceive of sequencing two samples for each individual cancer patient: one representing the inherited genome and the other the cancer genome in order to identify cancer specific genomic alterations. The ability to undertake genome-wide sequencing studies for thousands of cancer patients at base-pair resolution has had a profound impact on our understanding of the mutational landscape of cancers and the mechanisms underlying the disease.

Cancer is one of the leading cause of death worldwide, and it is well accepted that cells in the human body may acquire genomic changes (mutations). These mutations may result in uncontrolled cellular growth, suppression of cell-death signaling, promote angiogenesis amongst other key hallmark characteristics that lead to the development of cancers [1]. Over the last decade, two large consortia led the way in generating and analyzing NGS data from different cancer types. The Cancer Genome Atlas (TCGA) [2], predominantly using exome sequencing [3], and The International Cancer Genome Consortium (ICGC) [4], using whole genome sequencing, explored somatic mutations across thousands of cases from >30 cancer types. These consortia combined both sequencing approaches with transcriptional, methylation and protein analyses. Their cataloguing studies enabled a pan-cancer analysis [5] that explored the molecular characteristics of cancers, identifying recurring mutational patterns irrespective of the tissue of origin of the cancer. These studies generated a massive amount of knowledge about many cancer types, including the identification of (1) driver genes implicated in tumor development, (2) molecular cancer subtypes and (3) a mutational basis for targeted treatment options [6]. These consortia also have been instrumental in developing and providing methods and tools for the recording, analyses and sharing of multi-omic data on an international scale. One such analysis is the identification of mutational signatures, using information from hundreds to thousands of passenger mutations that might not cause cancer but can be a source of information about deficient cellular DNA damage repair mechanisms or patient exposure to carcinogens [7,8].

A major outcome of the NGS exploration of cancers has been the realization that high levels of inter-tumor and intra-tumor heterogeneity are key features of malignant cancers. Much of the current research efforts focus on how to identify patients whose cancers will respond to targeted treatments and how to negate the development of treatment resistance [9]. NGS of multiple samples, separated either spatially or temporally, from individual patients, have allowed the phylogenetic reconstruction of the evolution of mutational patterns and driver mutations arising in clonal compartments of cancers [10,11]. Such heterogeneity is known to support cancer cell survival in response to treatment due pre-existent or arising resistant clones. The major challenge remains how best to apply NGS to a single sample in space and time that addresses this well-recognized underlying complexity [12]. New technologies using NGS have lately been applied to liquid biopsy and showed potential to overcome some of the challenges of intra-tumor heterogeneity and further as a viable alternative to acquire material for genomic testing where biopsy or surgery are not an option, in advanced cancers. The sequencing of circulating cell-free DNA (cfDNA) obtained from blood promises a less invasive clinical testing process that has the potential to identify tumor mutations to guide treatment or identify mechanisms of treatment resistance; however, sensitivity remains an issue [13,14]. The American Society of Clinical Oncology and College of American Pathologists indicated that currently there is insufficient evidence for routine clinical use of cfDNA analysis and called for more studies to confirm clinical validity [15].

NGS has also revolutionized the study of the tumor transcriptome by identification of novel transcripts, fusion genes. Whole transcriptome sequencing also allow new insights into the tumor microenviroment using deconvolution methods to estimate the abundance of different cell types from a mixed cell population [16]. NGS has also revealed a tumorigenic role for non-coding RNAs with increasing evidence of their involvement in regulating gene expression affecting cancer cell plasticity and adding an extra layer of complexity in cancer biology. Non-coding RNAs have been described as regulatory molecules that mediate cellular processes, including chromatin remodeling, transcription, post-transcriptional modifications and signal transduction, which have the potential to be therapeutic targets [17]. However, there is still a great amount of research needed if this space to unravel the complex network non-coding RNAs in cancer biology.

In the research setting, new sequencing technologies that rely on the principals of NGS continue to be developed. For example: (1) ATAC-Seq (Assay for Transposase Accessible Chromatin with NGS) an approach for genome-wide chromatin accessibility profiling using hyperactive Tn5 transposase, which simultaneously cuts DNA and inserts sequencing adaptors [18]. (2) ChiP-Seq (chromatin immunoprecipitation combined with NGS) uses antibodies to select specific proteins or nucleosomes, to enrich for DNA fragments that are bound to these proteins or nucleosomes that are recovered and sequenced to provide a map of binding sites [19,20]. These are important tools to identify genome-wide changes in regulatory elements such transcription factor binding sites, enhancer and insulators regulating gene expression.

RNASeq together with single cell sequencing and spatial transcriptomics are bring new information allowing the study of subpopulations of cells and their relationships in the tumor microenvironment leading to unprecedented gain in knowledge in cancer biology [21]. The much anticipated single-molecule long-read sequencing technologies are beginning to be widely available as costs are reduced and platforms delivery stabilizes. Therefore, long-read sequencing is poised to deliver the next layer of new insights in cancer research by allowing the resolution of repeat sequences (a limiting ability of short read NGS), complex structural rearrangements and transcript isoform structure. Overall, the NGS revolution has allowed researchers to ask and answer questions about tumor biology that could not be performed previously. Developments in NGS and emerging novel technologies continue to change the cancer research landscape to advance our knowledge with the hope that in the near future we can further improve our ability to prevent, diagnose and treat cancers.
